# Estimating a Preference-Based Index from the Clinical Outcomes in Routine Evaluation–Outcome Measure (CORE-OM): Valuation of CORE-6D

**DOI:** 10.1177/0272989X12464431

**Published:** 2013-04

**Authors:** Ifigeneia Mavranezouli, John E. Brazier, Donna Rowen, Michael Barkham

**Affiliations:** Health Economics and Decision Science, School of Health and Related Research, University of Sheffield, Sheffield, UK (IM, JEB, DR); National Collaborating Centre for Mental Health, Centre for Outcomes Research and Effectiveness, Research Department of Clinical, Educational & Health Psychology, University College London, London, UK (IM); Centre for Psychological Services Research, University of Sheffield, Sheffield, UK (MB)

**Keywords:** condition specific, CORE-6D, CORE-OM, health state valuation, mental health, preference-based index, time trad-eoff

## Abstract

**Background**. The Clinical Outcomes in Routine Evaluation–Outcome Measure (CORE-OM) is used to evaluate the effectiveness of psychological therapies in people with common mental disorders. The objective of this study was to estimate a preference-based index for this population using CORE-6D, a health state classification system derived from the CORE-OM consisting of a 5-item emotional component and a physical item, and to demonstrate a novel method for generating states that are not orthogonal. **Methods**. Rasch analysis was used to identify 11 emotional health states from CORE-6D that were frequently observed in the study population and are, thus, plausible (in contrast, conventional statistical design might generate implausible states). Combined with the 3 response levels of the physical item of CORE-6D, they generate 33 plausible health states, 18 of which were selected for valuation. A valuation survey of 220 members of the public in South Yorkshire, United Kingdom, was undertaken using the time tradeoff (TTO) method. Regression analysis was subsequently used to predict values for all possible states described by CORE-6D. **Results**. A number of multivariate regression models were built to predict values for the 33 health states of CORE-6D, using the Rasch logit value of the emotional state and the response level of the physical item as independent variables. A cubic model with high predictive value (adjusted R^2^ = 0.990) was selected to predict TTO values for all 729 CORE-6D health states. **Conclusion**. The CORE-6D preference-based index will enable the assessment of cost-effectiveness of interventions for people with common mental disorders using existing and prospective CORE-OM data sets. The new method for generating states may be useful for other instruments with highly correlated dimensions.

Quality-adjusted life-years (QALYs) are increasingly used as the measure of benefit in economic evaluations of health care technologies and programs worldwide. Several preference-based measures have been developed aiming at the estimation of utility values that can be used for calculation of QALYs. Among the most widely used are the EuroQol-5D (EQ-5D),^1^ the SF-6D,^2^ and the HUI-3.^3^ All 3 measures are generic and can therefore be used for the assessment of interventions and programs targeted at different disease areas and patient populations.

However, generic measures may be less appropriate or sensitive in some medical conditions.^4,5^ Especially in the area of mental health, there are concerns that generic measures may lack sensitivity in capturing important elements of health-related quality of life (HRQoL), due to their focus on physical aspects of health (for example, 4 of 5 items of the EQ-5D capture physical aspects of HRQoL). This has led to proposals for the development of a preference-based measure specific to mental health that will be suitable for use across a wide range of mental health conditions.^6–8^ Currently, no such measure is available. A report examining the feasibility of incorporating patient-rated measures in mental health into a productivity measure for use in the United Kingdom identified the Clinical Outcomes in Routine Evaluation–Outcome Measure (CORE-OM) as a good candidate for this purpose.^9^

The CORE-OM is a patient-based instrument that is widely used in the United Kingdom to evaluate the effectiveness of psychological therapies in people with common mental disorders.^10,11^ It consists of 34 items, each with 5 levels of response (ranging from *not at all* to *most or all the time*), tapping 4 conceptual domains: subjective well-being, problems, functioning, and risk. The validity, reliability, and acceptability of the CORE-OM have been demonstrated across a wide range of practice settings.^12,13^ Based on these characteristics and given the arguments favoring the development of a preference-based measure specific to mental health, the CORE-OM was selected as the basis for constructing such a measure for use in common mental disorders.

Derivation of a preference-based measure from the CORE-OM requires a 3-step process: first, the development of a health state descriptive system; second, a valuation survey, in which respondents attach utility values to selected health states derived from the descriptive system; and third, modeling of the utility values leading to an algorithm that links all possible health states to utility values. Previous work has reported on the first stage of this process, that is, the construction of CORE-6D, a health state descriptive system derived from the CORE-OM.^14^ The primary objective of this article is to report on the later stages covering the development of an algorithm linking all health states described by CORE-6D with appropriate utility values, using the results of a valuation survey on CORE-6D health states and further modeling. A secondary objective is to examine an alternative method for generating health states when dimensions are highly correlated using the results of Rasch analysis.

## Methods

### The CORE-6D Health Descriptive System

CORE-6D is a 6-item health descriptive system consisting of a 5-item unidimensional emotional component and a physical item.^14^ Each item has 3 response levels: *never, only occasionally or sometimes*, and *often, most or all the time*. The system describes 3^6^ = 729 unique health states. The emotional component of CORE-6D was derived from the CORE-OM using predominantly Rasch analysis on a study sample of 400 people with common mental disorders selected randomly from a larger sample of 6610 patients presenting to National Health Service (NHS) primary care counseling services in the United Kingdom.^14^ Details of the original study sample of 6610 patients can be found in Evans and others.^15^ This sample included patients with a range of symptom severity and variable contact with different types of services: 3.8% of patients were concurrently attending secondary care or specialist services for a psychological problem, whereas another 9.9% had been in contact with such services for a psychological problem in the past. Moreover, patients with more severe symptoms could potentially be referred to secondary or tertiary services. The unidimensional emotional component of CORE-6D, combined with the physical item, creates a 2-dimensional scale, tapping emotional and physical symptoms in people with common mental disorders. The CORE-6D health state descriptive system is shown in Table 1.

**Table 1:** The CORE-6D Descriptive System

Emotional component		
1	I never feel terribly alone and isolated	0
	I feel terribly alone and isolated only occasionally or sometimes	1
	I feel terribly alone and isolated often, most or all the time	2

2	I never feel panic or terror	0
	I feel panic or terror only occasionally or sometimes	1
	I feel panic or terror often, most or all the time	2

3	I never feel humiliated or shamed by other people	0
	I feel humiliated or shamed by other people only occasionally or sometimes	1
	I feel humiliated or shamed by other people often, most or all the time	2

4	I am able to do most things I need to often, most or all the time	0
	I am able to do most things I need to only occasionally or sometimes	1
	I am not able to do the things I need to	2

5	I never make plans to end my life	0
	I make plans to end my life only occasionally or sometimes	1
	I make plans to end my life often, most or all the time	2

Physical health item		
6	I am never troubled by aches, pains, or other physical problems	0
	I am troubled by aches, pains, or other physical problems only occasionally or sometimes	1
	I am troubled by aches, pains, or other physical problems often, most or all the time	2

### Rasch Analysis

Rasch analysis is a statistical measurement approach for examining the relationship between people’s attributes (such as knowledge, quality of life, morbidity) and ordinal scales designed for the measurement of such attributes. It is based on the principles of the Rasch model,^16^ according to which the outcome of an encounter between a person and an item is exclusively governed by the product of the person’s ability (i.e., the person’s “amount” of the attribute) and the item’s difficulty (i.e., how much “quantity” of the attribute the item is able to capture).^17^ The model is a probabilistic form of Guttman scaling, a deterministic pattern that expects a strict hierarchical ordering of items (e.g., from low to high difficulty) such that if a person has affirmed an item of a given level of difficulty, then all items below that on the scale (i.e., easier items) should also be affirmed.^18^ The Rasch model relaxes this proposition by stating that if a more difficult item is affirmed, then there is a high probability that easier items will also be affirmed.^17^ The probability of a correct (affirmed) response to an item increases as the ability of a person increases, and the difficulty of the item decreases.^19^ Although Rasch analysis was originally developed for application in dichotomous items, the theory has been extended for the analysis of polytomous categorical scales.^20^

The Rasch model is underpinned by the principle of unidimensionality, meaning that items of a scale fitting the Rasch model capture one single attribute.^17^ Rasch analysis can convert ordinal scale scores into measurements of the attribute on a continuous scale with interval properties using a logit model^17,19,21^ and can assign individual items and persons on different points (or locations) along this scale, according to each item’s difficulty (reflected in the percentage of persons affirming an item) and each person’s ability (reflected in the percentage of items affirmed by the person).^19^ Each location along the scale corresponds to a Rasch model logit value, with higher values expressing more difficult items and more “able” persons (i.e., persons with higher amounts of the attribute). Assignment of persons to different points along the scale leads to generation of groups of persons with different levels of ability in the measured attribute.^19^

Rasch analysis has been successfully used as a tool for the development and refinement of unidimensional quality-of-life measures^21^ and more recently for item selection for the derivation of various condition-specific preference-based measures from existing ordinal scales.^22–25^

### Application of Rasch Analysis on the Emotional Component of CORE-6D

Rasch analysis was used at the development of CORE-6D to select the items forming its emotional component and to confirm the latter’s unidimensionality.^14^ The emotional component of CORE-6D comprises an ordinal scale of 5 items with 3 levels of response each. As shown on Table 1, each level of response gets an individual score (0-1-2); the total score is the sum of individual scores, ranging from 0 to 10, with higher scores indicating higher levels of emotional distress. Rasch analysis was used to convert respondents’ total scores on the emotional component of CORE-6D into interval scores on the Rasch model logit scale, with higher Rasch logit values indicating higher levels of emotional distress (and therefore more severe emotional health states). Persons with the same level of emotional distress had the same total ordinal score on the emotional component of CORE-6D and were therefore assigned the same Rasch model logit value.

### Selection of Health States for the Valuation Survey

The emotional component of CORE-6D can describe 3^5^ = 243 health states. However, because this component has been shown to be unidimensional,^14^ its items are not independent from each other, resulting in some item response combinations being implausible, for example, “I make plans to end my life often, most or all the time,” *and* “I never feel terribly alone and isolated.” Use of conventional statistical approaches for generating health states (such as orthogonal arrays) is not appropriate in this case because it is likely to generate implausible health states due to the high correlation between items. We have applied a novel method for generating health states, the Rasch vignette approach, to identify plausible health states amenable to valuation.^14^ This approach relies on the inspection of the item threshold map for the unidimensional emotional component, an output of Rasch analysis, which depicts the most likely item response combinations expected for each location across the Rasch model logit scale; this means that the map helps identify *one* most likely response combination for each level of emotional distress captured by the emotional component of CORE-6D, from mildest to most severe. These response combinations represent frequently observed health states experienced by people with common mental disorders across the continuum of severity of emotional distress, and therefore they describe actual, plausible health states. It must be noted that the item threshold map allows identification of *the most likely* (and thus plausible) health state at each location across the continuous Rasch scale; it does not depict *every* plausible health state described by a unidimensional scale. For each level of emotional symptom severity, there are several other plausible health states that are not depicted on the map, as they are less likely to be observed in the study population in comparison with the depicted state of that severity level.

**Figure 1:**
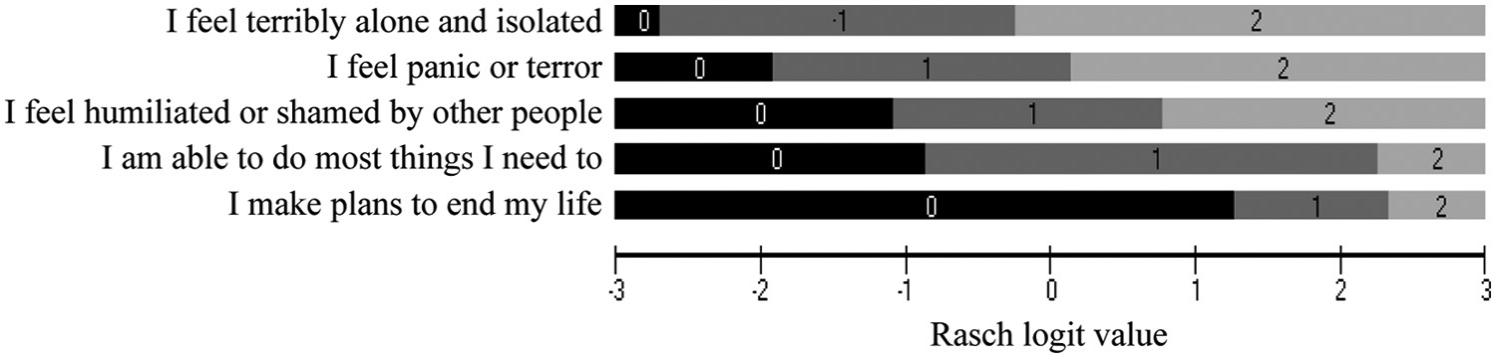
Rasch item threshold map of the emotional component of CORE-6D, from Mavranezouli et al., Quality of Life Research 2011; 20(3): 321-33, reprinted with kind permission from Springer Science + Business Media. 0 = never; 1 = only occasionally or sometimes; 2 = often, most or all the time; note that the fourth item is positively worded and therefore response levels are reversed.

Inspection of the Rasch item threshold map of the emotional component of CORE-6D in Figure 1 helped identify the most likely item response combinations across the continuum of the emotional symptom severity. Items have been ordered from the easiest to the most difficult, as indicated by their average location in the Rasch model. Shaded areas 0 (black), 1 (dark gray), and 2 (light gray) correspond to the 3 response levels, that is, *“never,” “only occasionally or sometimes,”* and *“most or all of the time,”* respectively, with the exception of the positively worded item, the response levels of which are reversed. The map allows prediction of the most likely response at each level of emotional symptom severity. For example, a person whose level of emotional distress corresponds to Rasch logit value +1 on the Rasch logit scale is expected to most likely respond 22210.

As illustrated in Table 2, 11 emotional health states (response combinations) were identified, each reflecting the most likely emotional state to be observed in a person with common mental disorders at a specific level of emotional symptom severity. These 11 emotional states represent only 4.5% of the 3^5^ = 243 potential health states described by the emotional component of CORE-6D but actually covered 37.1% of the response combinations obtained from the study sample (after excluding cases with 1 or more responses missing). To obtain the full CORE-6D state, each emotional health state needs to be combined with different response levels of the physical item. The 11 emotional health states selected by inspection of the item threshold map combined with the 3 response levels of the physical item of CORE-6D produce a 2-dimensional set of 11 × 3 = 33 health states that are frequently observed in the study population and, as such, are plausible. However, emotional health state 10 (22221) was not represented in the study sample (as shown in Table 2) and was therefore excluded from further consideration.

**Table 2:** Health States of the Emotional Component of CORE-6D as Identified by the Item Threshold Map and Frequency of Each Health State in the Study Sample

Item	Health State										
	1	2	3	4	5	6	7	8	9	10	11
I feel terribly alone and isolated	0	1	1	1	1	2	2	2	2	2	2
I feel panic or terror	0	0	1	1	1	1	2	2	2	2	2
I feel humiliated or shamed by other people	0	0	0	1	1	1	1	2	2	2	2
I am able to do most things I need to	0	0	0	0	1	1	1	1	1	2	2
I make plans to end my life	0	0	0	0	0	0	0	0	1	1	2
Frequency of each health state in the study sample (%)	5.3	5.9	6.2	5.0	5.6	2.7	2.7	1.5	1.5	0.0	0.6

Note: Adapted from Mavranezouli et al., Quality of Life Research 2011;20(3):321–33,^14^ with the kind permission from Springer Science + Business Media. 0 = *never*, 1 = *only occasionally or sometimes*, 2 = *often, most or all the time*. Note that the fourth item is positively worded and therefore response levels are reversed.

The remaining 10 emotional health states combined with the physical item at response level zero (never troubled by aches, pains, or other physical problems) were selected for valuation. In addition, and to assess the impact of physical functioning on utility values, 4 of these emotional states (including best state 00000, worst state 22222, and 2 intermediate states) were also combined with levels 1 and 2 of the physical item, so as to cover the full severity range captured by CORE-6D, thus producing another 8 CORE-6D health states. Intermediate emotional states 3 (11000) and 7 (22110) were chosen for this purpose, based on their relative frequency in the study sample (shown in Table 2) and their location coverage (range) on the item threshold map (shown in Figure 1). In total, 18 CORE-6D health states were selected for the valuation survey, plus 4 emotional health states with no reference to the physical item. Responses to the states describing only the emotional component of CORE-6D are analyzed elsewhere.^26^

Three card blocks were used in the valuation study. Every card block contained 8 cards, each describing a health state. Two of the card blocks included full CORE-6D health states. The third card block included 4 full CORE-6D health states and also 4 emotional health states, identical with the emotional components of the 4 CORE-6D states already included in this card block but without any reference to the physical item. CORE-6D state 222220 was included in all 3 card blocks. A sample of a health state card used in the valuation survey is presented in Table 3.

**Table 3:** Sample of a Health State Card Used in the Valuation Survey: Card Describing CORE-6D State 221101

• You feel terribly alone and isolated *often, most or all the time*
• You feel panic or terror *often, most or all the time*
• You feel humiliated or shamed by other people *only occasionally or sometimes*
• You are able to do most things you need to *only occasionally or sometimes*
• You *never* make plans to end your life
• You are troubled by aches, pains, or other physical problems *only occasionally or sometimes*

### Valuation Survey

A valuation survey using face-to-face interviews was carried out in South Yorkshire, UK, aiming at determining public preferences for a number of health states derived from CORE-6D. Selected health states were valued using the time tradeoff (TTO) technique, which asks respondents to trade HRQoL for life prolongment. More specifically, respondents are asked to choose either to live for a period of *t* years in a specified health state (*h_i_*) that is worse than full health or to shorten their life span to *x* years in full health, where *x* < *t*. The number of *x* years in full health is varied, until the point where the respondent is indifferent or switches preferences between the 2 alternatives. The utility value given to the state *h_i_* is then *x*/*t*.^27^

The version of TTO developed by the Measurement and Valuation of Health (MVH) group was used, including the visual props designed by this group.^28^ According to this protocol, respondents were first asked whether they preferred to live in a specified health state *h_i_* for *t* = 10 years after which they died or to die immediately. This question determined whether respondents valued the health state as better, worse, or equal to being dead. For health states considered better than death, the general TTO technique described earlier was used, with *t* = 10. For health states considered worse than being dead, respondents were asked to choose between life in the health state *h_i_* for *y* years followed by full health for *x* years after which they die (with *y* + *x* = 10) and immediate death. Years in full health (*x*) were varied concurrently with years in the health state (*y*) until the point at which respondents were indifferent or switched preferences between the 2 options. Valuations in the case of states considered worse than dead were estimated using the formula −*x*/10, following the same process with that reported at the TTO valuation of the UK EQ-5D,^28^ so that TTO values for states worse than dead were bounded by −1.

Interviews were conducted by trained and experienced interviewers from the Centre for Health and Social Care Research at Sheffield Hallam University. Respondents were selected using sampling from streets in both urban and rural areas with a mix of socioeconomic characteristics in the North of England using a comprehensive contact management system for names and addresses in the United Kingdom (AFD Names and Numbers version 3.1.25 database, AFD Software Limited, Ramsey, UK). Households in these areas received letters informing them that interviewers would be in their area, and interviewers then visited houses. Subsequently, all eligible and willing participants were interviewed in the respondent’s own home. The eligible population consisted of adults aged 18 years and older, who were considered by the interviewers to be cognitively able to participate in an interview. Addresses were visited up to 4 times on different days and times of the day before an address was considered a nonresponder. No financial reward was offered for participation in the survey. Ethical approval for the valuation survey was received by the ScHARR Research Ethics Committee at the University of Sheffield.

Respondents were first asked to self-complete EQ-5D and CORE-6D for their own health, so as to become familiar with the idea of describing states as well as with the items and response levels of CORE-6D. Subsequently, each respondent was given 1 of the 3 card blocks and undertook warm-up ranking and TTO tasks followed by TTO valuations of 8 health states. If, during the TTO valuations, it was made clear that a respondent did not understand the TTO task, the interview was terminated by the interviewer, and these partially completed interviews were not included in the data set for analysis. The following exclusion criteria were applied: respondents with 2 or fewer responses, respondents who valued the worst state higher than all other states, respondents who valued all states worse than being dead, and respondents who valued all states identically but lower than 1. Each interviewer started with a different card block with their first respondent and moved on systematically alternating card blocks in the same order in successive interviews; for example, the interviewer starting with card block 1 for the first respondent moved to card block 2 with the second respondent, then used card block 3 for the third respondent, then back to 1 with the fourth respondent, and so on. More details on the TTO process can be found in Gudex.^29^

All respondents first ranked and valued 4 states and subsequently ranked and valued the remaining 4 states in the card block. In the card block that contained 4 emotional health states without reference to the physical item and 4 full CORE-6D states, the emotional states were ranked and valued first, followed by ranking and valuation of CORE-6D states, so that responders were not aware of the presence of the physical item when valuing the 4 emotional states. In the other 2 card blocks, the 4 CORE-6D states that were ranked and valued first were chosen at random. Because of the nature of some item responses (e.g., I make plans to end my life), respondents were informed in the cover letter and information sheet that the interview was about common mental and physical health problems. In the information sheet and in a thank you note left at the end of the interview, all respondents were strongly recommended that they seek appropriate professional support either from their general practitioner or from a professional agency such as the Samaritans (contact details provided) if the interview raised personal issues for them. Respondents were also asked a number of background questions covering health, demographic, and socioeconomic characteristics and how difficult they found the valuation tasks.

### Modeling TTO Values for All CORE-6D Health States Using Rasch Analysis

The standard approach for modeling utility values has been by creating dummy variables for each level of every dimension of an instrument^2,30^ and regressing these onto the health state values (obtained using TTO or standard gamble). However, this approach was not appropriate here because the highly correlated items of the emotional component of CORE-6D were expected to produce significant, multiple interaction effects, and consideration of all possible interactions across different response levels of different items would require complex regression models as well as valuation of a large number of health states to predict TTO values for all health states of the instrument. This can be avoided using an alternative method described by Young and others^31^ that uses the relationship between the Rasch model logit value and the respective TTO value of a health state of a unidimensional measure to predict TTO values for all potential states of the measure.

Nevertheless, this new method alone was not adequate for the estimation of TTO values for CORE-6D; this is because CORE-6D is a 2-dimensional scale, consisting of a unidimensional emotional component and a physical item. To predict TTO values for all health states described by CORE-6D taking into account the effect of the physical item, we adopted a hybrid approach: We used as a basis the methodology described by Young and others^31^ that is appropriate for the prediction of TTO values in the case of unidimensional measures such as the emotional component of CORE-6D, and also created dummy variables to represent the different severity levels of the physical item, which is a standard approach used for multidimensional measures.^2,30^ Consequently, a series of regression analyses was undertaken on mean level data (i.e., on the mean TTO values obtained for each of the 18 health states included in the valuation survey, rather than on the individual TTO values obtained from each respondent in the survey) to explore the relationship between the TTO value for each health state considered in valuation and

1. the respective Rasch model logit value corresponding to the emotional component of the health state, as identified in previously undertaken Rasch analysis;
2. the response level (0, 1, or 2) of the physical item of the health state, modeled in the form of 2 dummy dichotomous variables, one for response level 1 and one for response level 2.

A number of regression models were fitted, including simple linear, quadratic, and cubic forms, to reflect potential nonlinearities in the relationship. Additional models that considered, in combination with the above, the potential (multiplicative) interaction between the emotional component of CORE-6D and the physical item (also considering linear, quadratic, and cubic relationship) were tested. Model fit was compared using the coefficient of determination (i.e., the adjusted *R* ^2^) and the root mean squared error (RMSE) at the health state level. The model with the best fit was selected to predict mean TTO values for all health states described by CORE-6D based on their respective Rasch model logit value and the response level of the physical item.

## Results

### Valuation Survey: Respondents’ Characteristics

The valuation survey was conducted on 225 respondents, a response rate of 45.7% for respondents answering their door at the time of interview. The study achieved a completion rate of 99.7% for all 18 health states included in the TTO valuations considered in this study (4 missing TTO values). Characteristics of all respondents included in the analysis are presented in Table 4, which allows comparison of the study sample to the general population in South Yorkshire and England. The study sample had a higher average age; a higher proportion of women, homeowners, and retired individuals; and a lower proportion of employed/self-employed individuals. A large proportion of respondents reported that they found the rank (35.1% of respondents) and TTO (40.9% of respondents) tasks either *very difficult* or *rather difficult*, and this likely includes both respondents who found completion of the task complex and respondents who found the decisions involved challenging. Finding a task difficult does not convey a lack of understanding, as no respondents met the set exclusion criteria that indicated no understanding of the TTO task. Moreover, interviewers reported that it was doubtful (according to their expert judgment) whether the respondent understood the rank and TTO tasks in just 5.8% and 4.9% of the interviews, respectively.

**Table 4:** Characteristics of Respondents in the Valuation Survey and Comparison with Population Characteristics for South Yorkshire and England

	Respondents (*n* = 225)	South Yorkshire^a^	England^a^
Mean age (SD)	48.86 (17.16)	—	—
Age distribution (%)			
18–40	32.7	41.2	41.6
41–65	48.0	39.1	39.1
>65	19.3	19.7	19.3
Female (%)	58.7	51.2	51.3
Married/partner (%)	69.8	NA	—
Employed or self-employed (%)	51.3	56.1	60.9
Unemployed (%)	3.1	4.1	3.4
Long-term sick (%)	5.4	7.7	5.3
Full-time student (%)	5.4	7.5	7.3
Retired (%)	22.3	14.4	13.5
Own home outright or with a mortgage (%)	81.0	64.0	68.7
Renting property (%)	20.0	36.0	31.3
Secondary school is highest level of education (%)	37.9	NA	—
Average EQ-5D score (SD)	0.83 (0.28)	NA	0.86 (0.23)^b^
Time tradeoff (TTO) completion rate (%)	99.7	—	—
Respondent found first rank valuation task very or rather difficult (%)	35.1	—	—
Respondent found first TTO valuation task very or rather difficult (%)	40.9	—	—
Interviewer doubted whether respondent understood first rank task	5.8	—	—
Interviewer doubted whether respondent understood first TTO task	4.9	—	—

a. Statistics for South Yorkshire Health Authority and for England in the Census 2001. Questions used in this study and the census are not identical. The census includes persons aged 16 and older, whereas this study surveyed persons aged 18 and older only. Age distribution is here reported as the percentage of all adults aged 18 and older.

b. Interviews conducted in the Measurement and Valuation of Health (MVH) study.^32^

### TTO Values Obtained from the Valuation Survey

The TTO values obtained from the valuation survey are reported in Table 5 and Table 6. Table 5 provides descriptive statistics for the health state values obtained for each health state. It can be seen that the mean TTO values range from 0.96 (best state 000000) to 0.10 (worst state 222222). Table 6, which shows responses by card block, demonstrates the changes in obtained TTO values with increasing severity of physical and emotional symptoms: Moving to states with more severe physical symptoms (i.e., increasing the response level of the physical item), while keeping the emotional health state unchanged, results in a decrease in the average TTO value; similarly, moving to states with more severe emotional symptoms (i.e., moving from emotional state 00000 to emotional state 22222), while keeping the response level of the physical item intact, also results in a decrease in the average TTO value. There is only one inconsistency to this pattern, observed in states 100000 and 110000; in this case, the mean TTO value increased by a small and nonsignificant amount (from 0.87 to 0.88, respectively) despite the increase in the emotional symptom severity. This inconsistency can be explained by the fact that these health states were included in different card blocs and hence were valued by different respondents.

**Table 5:** Time Tradeoff (TTO) Values by Health State Obtained in the Valuation Survey

CORE-6D Health State	TTO Value								
	N	Mean	SD	Minimum	Percentile 25	Median	Percentile 75	Maximum	Mode
000000	75	0.96	0.13	0.08	0.99	1.00	1.00	1.00	1.00
000001	75	0.93	0.14	0.33	0.93	1.00	1.00	1.00	1.00
000002	76	0.82	0.32	−0.93	0.78	0.93	1.00	1.00	1.00
100000	74	0.87	0.22	0.08	0.84	1.00	1.00	1.00	1.00
110000	75	0.88	0.25	−0.73	0.85	1.00	1.00	1.00	1.00
110001	76	0.86	0.27	−0.93	0.80	0.96	1.00	1.00	1.00
110002	75	0.74	0.31	−0.83	0.57	0.83	1.00	1.00	1.00
111000	74	0.79	0.29	−0.23	0.69	0.93	1.00	1.00	1.00
111100	74	0.76	0.33	−0.40	0.53	0.93	1.00	1.00	1.00
211100	75	0.66	0.35	−0.63	0.50	0.73	1.00	1.00	1.00
221100	76	0.57	0.44	−0.93	0.45	0.63	0.93	1.00	1.00
221101	74	0.49	0.47	−0.88	0.30	0.50	0.88	1.00	1.00
221102	74	0.40	0.49	−0.93	0.14	0.44	0.83	1.00	1.00
222100	74	0.47	0.43	−0.93	0.20	0.50	0.84	1.00	1.00
222110	75	0.38	0.45	−0.98	0.08	0.44	0.70	1.00	1.00
222220	225	0.23	0.52	−0.98	0.00	0.30	0.53	1.00	1.00
222221	74	0.21	0.50	−0.93	−0.08	0.23	0.50	1.00	1.00
222222	75	0.10	0.53	−0.93	−0.33	0.10	0.48	1.00	1.00

**Table 6:** Mean Time Tradeoff Values for Each CORE-6D Health State Included in Valuation Survey by Severity of Emotional and Physical Symptoms^a^

CORE-6D	Response Level of Physical Item		
Emotional component	0	1	2
00000	0.96 (0.13)	0.93 (0.14)	0.82 (0.32)
10000	0.87 (0.22)		
11000	0.88 (0.25)	0.86 (0.27)	0.74 (0.31)
11100	0.79 (0.29)		
11110	0.76 (0.33)		
21110	0.66 (0.35)		
22110	0.57 (0.44)	0.49 (0.47)	0.40 (0.49)
22210	0.47 (0.43)		
22211	0.37 (0.45)		
22221			
22222	0.23 (0.52)	0.21 (0.50)	0.10 (0.53)

a. Standard deviation is in parentheses. Each card bloc is highlighted in a different shade; all respondents valued state 222220, shaded in black.

### Modeling TTO Values of CORE-6D Health States Using the Respective Rasch Model Logit Values and the Response Level of the Physical Item

The Rasch model logit values for each emotional health state were rescaled and anchored at 0.96 and 0.23, which were the observed mean TTO values corresponding to the CORE-6D health states with the best and worst emotional states 00000 and 22222, respectively, and response level zero for the physical item, obtained from the valuation survey. To predict TTO values for the 33 CORE-6D health states (formed by combining the emotional states depicted in the item threshold map with the 3 response levels of the physical item), a number of mean level regression models were explored using as independent variables the Rasch model rescaled logit value (assuming simple linear, quadratic, and cubic relationships) and 2 dummy variables accounting for the response levels 1 and 2 of the physical item.

The following model specifications were tested:

Model 1, simple linear relationship: *y =* α *+* β*_1_R +* γ*_1_P_1_ +* γ*_2_P_2_*
Model 2, quadratic relationship: *y* = α + β*_2_R^2^ + γ_1_P_1_ + γ_2_P_2_*
Model 3, cubic relationship: *y* = α + β*_3_R^3^ + γ_1_P_1_ + γ_2_P_2_*
Model 4, quadratic relationship: *y* = α + β*_1_R + β_2_R^2^ + γ_1_P_1_ + γ_2_P_2_*
Model 5, cubic relationship: *y* = α + β*_1_R + β_3_R^3^ + γ_1_P_1_ + γ_2_P_2_*
Model 6, cubic relationship: *y* = α + β*_2_R^2^ + β_3_R^3^ + γ_1_P_1_ + γ_2_P_2_*
Model 7, cubic relationship: *y* = α + β*_1_R + β_2_R^2^ + β_3_R^3^ + γ_1_P_1_ + γ_2_P_2_*

where *y* is the mean predicted TTO value, *R* is the Rasch model rescaled logit value, *P_1_* is a dummy variable for response level 1 of the physical item (I have been troubled by aches, pains, physical problems only occasionally or sometimes), *P_2_* is a dummy variable for response level 2 of the physical item (I have been troubled by aches, pains, physical problems often, most or all the time), α is the constant, and β_*i*_ and γ_*i*_ are regression coefficients.

The regression coefficients and goodness-of-fit statistics for all 7 models are shown in Table 7. The adjusted *R* ^2^ statistics varied from 0.773 (model 3) to 0.990 (model 7). In all models, dummy variable *P_1_* was nonsignificant. In model 7, the level of significance was only slightly greater than 0.05 (0.069). Based on having the lowest RMSE statistics of 0.0275, the largest model that contained linear, quadratic, and cubic terms for the logit value and both physical dummies (model 7) was selected for the prediction of TTO values for the 33 CORE-6D health states; this model had also the best fit in terms of the adjusted *R* ^2^. The additional models that considered interaction terms between the emotional component and the physical item of CORE-6D did not appear to offer any improvement in the model fit compared with the selected model 7; in all of these additional models, the interaction terms were not significant. These findings suggest that a simple additive model was adequate to capture the relationship between the TTO values, on the one side of the equation, and the Rasch logit value of the emotional component as well as the physical dummy variables, on the other.

**Table 7:** Regression Models for Prediction of Mean Time Tradeoff Values (*y*) from Rasch model Rescaled Logit Values (R) after Adding 2 Dummy Variables (*P*_1_, *P*_2_) to Account for the Response Level of the Physical Item (Response Levels 1 and 2, Respectively)

Model	α	β_1_	β_2_	β_3_	γ_1_	γ_2_	Adjusted *R* ^2^	RMSE
Model 1: *y =* α *+* β*_1_R +* γ*_1_P_1_ +* γ*_2_P_2_*	0.008 (0.833)	1.057 (0.000)			−0.044 (0.189)	−0.151 (0.000)	0.961	0.0533
Model 2: *y =* α *+* β*_2_R^2^ +* γ*_1_P_1_ +* γ*_2_P_2_*	0.302 (0.000)		0.844 (0.000)		−0.070 (0.219)	−0.177 (0.006)	0.886	0.0916
Model 3: *y =* α *+* β*_3_R^3^ +* γ*_1_P_1_ +* γ*_2_P_2_*	0.416 (0.000)			0.779 (0.000)	−0.085 (0.284)	−0.193 (0.025)	0.773	0.1292
Model 4: *y =* α *+* β*_1_R +* β*_2_R^2^ +* γ*_1_P_1_ +* γ*_2_P_2_*	−0.130 (0.100)	1.585 (0.000)	−0.443 (0.056)		−0.029 (0.329)	−0.137 (0.000)	0.969	0.0478
Model 5: *y =* α *+* β*_1_R +* β*_3_R^3^ +* γ*_1_P_1_ +* γ*_2_P_2_*	−0.108 (0.072)	1.388 (0.000)		−0.282 (0.025)	−0.028 (0.329)	−0.135 (0.000)	0.972	0.0452
Model 6: *y =* α *+* β*_2_R^2^ +* β*_3_R^3^ +* γ*_1_P_1_ +* γ*_2_P_2_*	0.099 (0.002)		2.624 (0.000)	−1.758 (0.000)	−0.029 (0.170)	−0.137 (0.000)	0.985	0.0331
Model 7: *y =* α *+* β*_1_R +* β*_2_R^2^ +* β*_3_R^3^ +* γ*_1_P_1_ +* γ_2_P_2_	0.366 (0.004)	−1.695 (0.022)	5.712 (0.000)	−3.446 (0.000)	−0.033 (0.069)	−0.141 (0.000)	0.990	0.0275

Note: RMSE = root mean squared error. *P* values are in parentheses.

**Figure 2:**
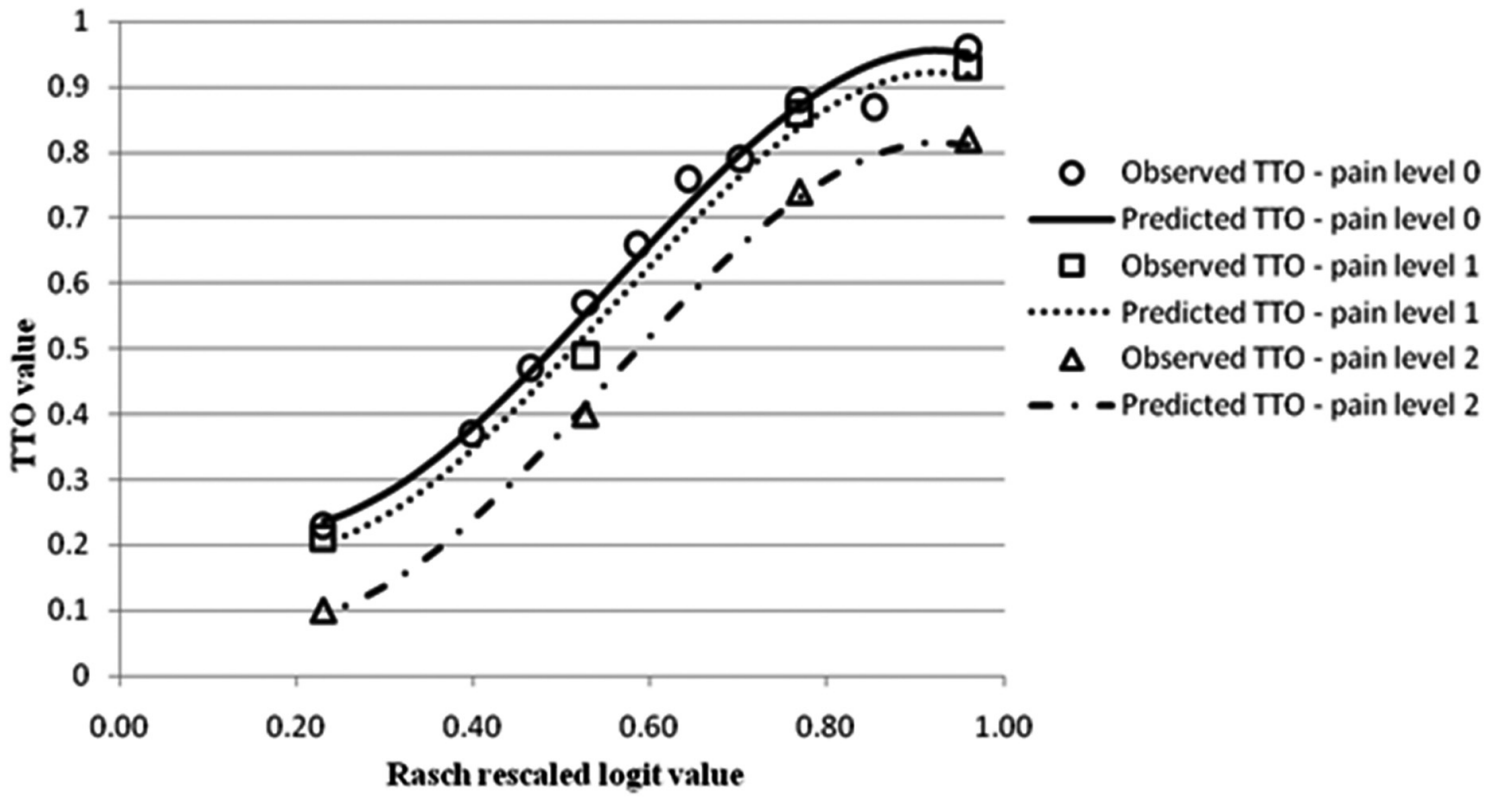
Mean observed (from the valuation survey) and modeled (based on regression model 7) time tradeoff values by Rasch rescaled logit value. Modelled time trade-off (TTO) values are predicted using the Rasch rescaled logit value of the emotional health state and the response level of the physical item ‘I am troubled by aches, pains, physical problems’ (level 0 = never; level 1 = only occasionally or sometimes; level 2 = often, most or all the time).

Given that emotional health states with the same total (ordinal) score correspond to the same Rasch logit value, it is possible to predict TTO values for all CORE-6D health states, based on their total emotional component score and the response level of the physical item. Figure 2 allows the comparison between actual mean TTO values obtained from the valuation survey for the selected CORE-6D health states and predicted TTO values for all potential health states described by CORE-6D, derived from the regression model 7. The *x*-axis of the graph represents Rasch rescaled logit values that cover the full severity range of all potential emotional health states described by CORE-6D. The *y*-axis depicts TTO values. There are three lines on the graph, one for each level of the physical item. The 3 lines have an s-shape reflecting the cubic relationship between the Rasch logit scale and the TTO health state value.

Table 8 provides the modeled TTO values for all potential CORE-6D health states as estimated using the regression model 7. Estimation of the TTO value of each health state is based on the total score of the emotional component of the state and the response level of the physical item. An SPSS syntax file that allows calculation of CORE-6D TTO values from CORE-OM data is available from the corresponding author on request.

**Table 8:** Modeled Mean Time Tradeoff Values for All CORE-6D Health States, Based on the Total Score of the Emotional Component of the State and the Response Level of the Physical Item, Using Regression Model 7

CORE-6D Total Score of Emotional Component	Response Levels of Physical Item		
	0	1	2
0	0.95	0.92	0.81
1	0.94	0.90	0.80
2	0.87	0.84	0.73
3	0.80	0.77	0.66
4	0.72	0.69	0.58
5	0.64	0.61	0.50
6	0.55	0.52	0.41
7	0.47	0.43	0.32
8	0.38	0.35	0.24
9	0.30	0.26	0.16
10	0.24	0.20	0.10

## Discussion

This article describes the development of a new preference-based measure specific to patients with common mental disorders, using a novel methodology that is based predominantly on Rasch analysis. Rasch analysis was used for the development of the unidimensional emotional component of CORE-6D, the identification of plausible emotional health states that were subsequently considered in the valuation survey, and the generation of modeled TTO values for all health states of CORE-6D by estimating the relationship between the Rasch model logit values of the emotional component and the mean observed TTO values of the CORE-6D states included in the valuation survey using regression analysis. The characteristics of the selected cubic model (RMSE = 0.0275 and adjusted *R* ^2^ = 0.990) compare very favorably with regression models described in similar modeling studies, where the RMSE was typically greater than 0.05 and the adjusted *R* ^2^ was less than 0.6.^2,30,33–35^ Extra regression models that considered multiplicative interaction between the physical item and the emotional component of CORE-6D did not offer any improvement in the model fit compared with the selected model, thus suggesting that a simple additive model was adequate.

This finding supports an assumption that the impact of different dimensions on preferences is additive. If the assumption holds, inclusion or exclusion of a dimension should lead to no significant change in the coefficients of the other dimensions in the classification. However, this was not found in another study where a pain dimension was added to an asthma-specific utility measure, the AQL-5D.^36^ This resulted in the coefficients of 2 of the other dimensions being significantly changed. However, the case of AQL-5D is different because the other dimensions of the measure were primarily concerned with physical health and so were less independent from a pain dimension than the emotional component of CORE-6D.

The methodology presented in this article was dictated by the high correlation across the CORE-6D items, which precluded the use of standard statistical approaches for generating health states such as orthogonal arrays, as these would likely result in the selection of implausible health states. In terms of modeling TTO values, use of the standard approach by creating dummy variables in regression analysis by creating dummy variables for each level of every item of the measure in regression analysis^2,30^ would have required far more states to be valued. In contrast, use of Rasch analysis allowed identification of plausible health states for valuation and is a more efficient solution for modeling TTO values. Our study successfully developed a mixed approach for modeling TTO values by combining the Rasch-based approach reported by Young and others^31^ with the standard approach,^2,30^ used to account for the different severity levels of the physical item of CORE-6D. The generation of plausible health states for valuation has been validated in a separate data set of people with common mental disorders.^14^ Future work should aim to validate our findings from the valuation survey and the selected regression model using a different sample of the general population.

The methods proposed in this article for the derivation of new preference-based measures from existing instruments are appropriate to apply to measures with highly correlated dimensions, in order to overcome issues that would arise from use of standard approaches, such as the generation of implausible health states and the need for considering multiple interaction effects when modeling TTO values. Our suggested methods are likely most applicable to condition-specific measures that have a narrow scope, for example, by focusing mainly on symptoms or one aspect of patients’ HRQoL. In such cases, the dimensionality of the existing measure should be examined at an initial stage of the process; exploratory factor analysis can be used for this purpose, to give an indication of the extent of unidimensionality and the number of dimensions covered by the existing instrument.^37^ If the instrument is found to have a largely unidimensional component or highly correlated dimensions, then Rasch analysis can be used to select items in order to construct a unidimensional new measure (or 1 or more independent unidimensional components) and to select plausible health states for valuation.^14^ Subsequently, if the new health state classification comprises a unidimensional scale, then the approach described by Young and others^31^ can be adopted to predict TTO values for all potential health states using the results of Rasch analysis. If, on the other hand, the new health state classification comprises a multidimensional measure that encompasses 1 or more unidimensional components, then our hybrid approach can be used for modeling TTO values following the valuation of plausible health states.

The valuation of CORE-6D followed the MVH group TTO protocol that was developed for the valuation of EQ-5D.^28,30^ Adoption of this protocol permits comparability with the EQ-5D and meets the requirements of the National Institute for Health and Clinical Excellence (NICE) in England and Wales, according to which, when an alternative to EQ-5D is used, the same methods of valuation should be adopted.^38^ However, it is acknowledged that the MVH group TTO protocol suffers from a number of limitations,^39^ including the effect of respondents’ age on valuations.^28,40^ It could be argued that framing the valuation statements using a 10-year time horizon may feel too generous for older respondents and yet too short for younger ones. Further exploration of the impact of age on health state valuations, however, suggests that differences in valuations between young and old respondents would have still been observed if respondents’ life expectancy had been used rather than a fixed time horizon of the valuation statements.^41,42^ Other criticisms of the MVH group TTO protocol relate to the procedure for the valuation of states that are worse than death, including the apparently unrealistic scenario of moving from poor health to full health, the different tradeoff procedures between valuation of states worse than death and that of states better than death, and the monotonic transformation of values of states considered worse than death so that values are bounded by −1.^39^ Further analysis of the strengths and weaknesses of the TTO task and controversial issues relating to the valuation protocol of EQ-5D are outside the scope of this article; for an overview of the issues, see Rowen and Brazier.^43^

The new measure was derived from the CORE-OM using predominantly Rasch analysis on a sample of people with common mental disorders presenting to NHS primary care counseling services in the United Kingdom. It could be argued that people presenting to a primary care setting have a lower burden of disease and thus their condition is not representative of the full spectrum of common mental disorders in the community; consequently, CORE-6D may not be well targeted to the intended patient population (i.e., all patients with common mental disorders, regardless of their level of symptom severity). In practice, however, primary care is currently the dominant service provider for people with common mental disorders in the United Kingdom, and the choice between primary and secondary care is more related to access issues determining the pathway into the service rather than to the patients’ level of symptom severity. In support of this view, comparison of the results between the study that provided the primary care data set used for the development of CORE-6D^15^ and a similar study that included patients from secondary and specialist services only^10^ demonstrated that a number of findings, including, for example, the percentage of patients scoring above the clinical threshold at intake, were very similar between the 2 studies, “suggesting the robustness of certain parameters to changes of setting and cohort.”^15^ We therefore argue that the new measure is well suited to reflect HRQoL aspects of patients in the full spectrum of common mental disorders.

One limitation of the new measure is that it is suitable only for common mental disorders, such as depression and anxiety. The CORE-OM has not been designed for use in other mental disorders such as schizophrenia, bipolar disorder, personality disorders, and so forth. Consequently, CORE-6D cannot be used for the estimation of QALYs at the evaluation of interventions targeted at mental disorders other than depression and anxiety and therefore cannot be used as a generic mental health preference-based measure. Nonetheless, common mental disorders constitute the most prevalent group of mental disorders in the United Kingdom, experienced by 16.2% of people aged 16 to 64 years in England (for comparison, psychotic disorders are experienced by 0.4% of this population).^44^

Another limitation of CORE-6D is that it largely focuses on emotional symptoms, as it includes 5 emotional items and only 1 physical item. The composition of CORE-6D reflects the structure of the CORE-OM (from which CORE-6D was derived), which is a measure primarily designed for the monitoring of emotional, rather than physical, symptoms. Inclusion of 1 physical item in CORE-6D allows a rather crude representation of physical symptoms, which, nevertheless, enables the assessment and valuation of both emotional and physical dimensions of HRQoL in people with common mental disorders.

Compared with generic preference-based measures, condition-specific ones, such as CORE-6D, are expected to be more relevant and sensitive to the condition they have been designed for; on the other hand, they are characterized by a number of limitations, such as their inability to capture side effects of treatment and comorbidities, and the distortions created by focusing effects.^5^ The role of generic and condition-specific preference-based measures has been (and still is) an important subject of debate.^45–48^ Use of condition-specific preference-based measures raises concerns regarding their comparability to generic measures in the wider resource allocation context, although it has been argued that comparability across different measures can be improved if utility values are obtained using the same valuation technique, on a scale with common anchors (full health and death), and elicited from the same population.^27^

In the area of mental health, use of CORE-6D may be more suitable than the use of generic preference-based measures such as EQ-5D, SF-6D, and HUI-3. Indeed, with 5 of its 6 items representing emotional aspects of HRQoL, CORE-6D is likely more sensitive in capturing HRQoL changes in people with mental health disorders, compared, for example, with the generic EQ-5D, which consists of 4 items on physical health (mobility, self-care, usual activities, and pain/discomfort) and 1 mental health item (anxiety/depression).^1^ Similarly, HUI-3 contains 6 physical health attributes (vision, hearing, speech, ambulation, dexterity, and pain), 1 attribute on cognition, and only 1 on emotion.^3^ SF-6D, on the other hand, although generic, is somewhat more balanced between physical and emotional aspects of HRQoL, with 3 exclusively physical health dimensions (physical functioning, bodily pain, and vitality), 1 pure mental health dimension, and 2 dimensions relating to both physical and mental health (role limitations and social functioning).^2^

Probably because of their focus on physical aspects of HRQoL, generic measures have been reported to be less responsive in measuring HRQoL in patients with mental health problems.^6,49–53^ In addition, as the majority of their items are irrelevant to such populations, generic measures are often unacceptable to individuals with mental health problems.^54^ The unsuitability of generic measures for measurement of HRQoL changes in people with mental disorders as well as the unacceptability of generic measures to such populations may explain the findings of a systematic review of outcome measurement in psychiatric research and practice, according to which only a negligible portion of randomized controlled trials conducted in psychiatric research used generic measures.^54^ The same study concluded that “there is no robust research evidence to support the value [of generic measures] as routine measures of outcome in psychiatric settings.” In contrast, the CORE-OM (and thus CORE-6D) is a measure of outcome for common mental disorders that is widely used in clinical practice in the United Kingdom; moreover, it is acceptable to both patients and health care professionals and is also freely available to users.^10^ Therefore, the development of CORE-6D will allow broader conduct of cost-utility analyses in the area of mental health, especially in studies that include the CORE-OM but no generic preference-based measures.

The appropriateness and sensitivity of CORE-6D is currently being assessed as a next step of this study, with the new measure being compared with generic measures such as EQ-5D and SF-6D in populations of people with common mental disorders. Preliminary findings suggest that CORE-6D performs comparably to EQ-5D and SF-6D in terms of responsiveness and has higher discriminative ability across different severity groups. Given the routine use of the CORE-OM in the clinical monitoring of people with common mental disorders in the United Kingdom, the preference-based CORE-6D is expected to contribute to the wider assessment of the cost-effectiveness of interventions for common mental disorders using existing and prospective CORE-OM data sets.
